# Oral ambroxol increases brain glucocerebrosidase activity in a nonhuman primate

**DOI:** 10.1002/syn.21967

**Published:** 2017-03-17

**Authors:** Anna Migdalska‐Richards, Wai Kin D. Ko, Qin Li, Erwan Bezard, Anthony H. V. Schapira

**Affiliations:** ^1^ Department of Clinical NeurosciencesInstitute of Neurology, University College LondonNW3 2PFUnited Kingdom; ^2^ Motac NeuroscienceManchesterUnited Kingdom; ^3^ Institute of Laboratory Animal Sciences, China Academy of Medical SciencesBeijing CityPeople's Republic of China; ^4^ University de Bordeaux, Institut des Maladies Neurodégénératives, UMR 5293BordeauxF‐33000France; ^5^ CNRS, Institut des Maladies Neurodégénératives, UMR 5293BordeauxF‐33000France

**Keywords:** ambroxol, glucocerebrosidase, Parkinson disease, nonhuman primate

## Abstract

Mutations in the glucocerebrosidase 1 (*GBA1*) gene are related to both Parkinson disease (PD) and Gaucher disease (GD). In both cases, the condition is associated with deficiency of glucocerebrosidase (GCase), the enzyme encoded by *GBA1*. Ambroxol is a small molecule chaperone that has been shown in mice to cross the blood‐brain barrier, increase GCase activity and reduce alpha‐synuclein protein levels. In this study, we analyze the effect of ambroxol treatment on GCase activity in healthy nonhuman primates. We show that daily administration of ambroxol results in increased brain GCase activity. Our work further indicates that ambroxol should be investigated as a novel therapy for both PD and neuronopathic GD in humans.

## 1 Introduction

Glucocerebrosidase (GCase) is an enzyme that catalyses the breakdown of glycolipid glucocerebroside to ceramide and glucose, which is encoded by the glucocerebrosidase 1 (*GBA1*) gene (Beutler, 1992). *GBA1* mutations have been implicated in both Gaucher disease (GD) and Parkinson disease (PD). Gaucher disease is the most common lysosomal disorder caused by homozygous *GBA1* mutations whereas PD is the second most common neurodegenerative disorder after Alzheimer disease. The risk of developing PD is 20–30 times higher in GD patients and carriers than in the general population. Further, current estimates predict that ∼5–10% of PD patients carry a *GBA1* mutation (PD‐*GBA1*), although this figure is much greater in PD patients of Ashkenazi origin (Bultron et al., 2010; Migdalska‐Richards and Schapira, 2016; Sidransky et al., 2009).

The mechanism by which *GBA1* mutations increase PD risk is currently unknown, but given that pathological manifestations are identical in PD‐*GBA1* and idiopathic PD patients, it is predicted that, as in idiopathic PD, accumulation of alpha‐synuclein and dopaminergic neuron loss in the substantia nigra are the key components for PD‐*GBA1* (Migdalska‐Richards and Schapira, 2016; Wang et al., 2015). The existence of a reciprocal relationship between GCase activity and alpha‐synuclein levels has recently been shown both in cell models and in chemically and genetically induced *Gba1* mouse models (Migdalska‐Richards and Schapira, 2016). Moreover, GCase activity and idiopathic PD have recently been linked by the identification of significant reduction in GCase activity in several brain regions from these Parkinson patients (Gegg et al., 2012; Murphy and Halliday, 2014).

The increasing evidence highlighting the significance of GCase deficiency in both PD‐*GBA1* and idiopathic PD patients suggests that treatments that increase GCase might be advantageous to PD patients both with and without *GBA1* mutations. To this end, we have recently investigated a small molecular chaperone, ambroxol hydrochloride (ambroxol), in wild‐type mice, in transgenic *Gba1* mice carrying a heterozygous L444P mutation, and in transgenic mice overexpressing human alpha‐synuclein (SNCA). We showed that ambroxol is capable of crossing the blood‐brain barrier, leads to a significant increase in GCase activity in wild‐type and transgenic mice, and is able to decrease alpha‐synuclein and phosphorylated alpha‐synuclein protein levels in transgenic mice overexpressing SNCA (Migdalska‐Richards et al., 2016). Here, we extend this work by analyzing the effect of ambroxol treatment on GCase activity in healthy nonhuman primates.

## 2 Material and methods

### 2.1 Cynomolgus monkeys

Experiments were carried out in accordance with European Communities Council Directive of 24 November 1986 (86/609/EEC) revised in 2010 (2010/63/EU) for the care of laboratory animals following acceptance of the study design by the Institute of Lab Animal Science (Chinese Academy of Science, Beijing, China) IACUC in an AAALAC‐accredited facility. Three adult male cynomolgus monkeys (*Macaca fascicularis*, Xierxin, Beijing, PRC) were housed in individual primate cages under controlled conditions of humidity, temperature, and light (12‐hr light/12‐hr dark cycle, lights on at 8.00 a.m.); food and water were available ad libitum. Animal care was supervised by veterinarians experienced in the husbandry and care of nonhuman primates.

### 2.2 Ambroxol hydrochloride administration

Nonhuman primates underwent oral chronic treatment with vehicle (*n* = 1), ambroxol (Sigma‐Aldrich) 22.5 mg/day (*n* = 1), and ambroxol 100 mg/day (*n* = 1) for 28 days. They were clinically followed during the experiment timescale for general health, appearance, motor, and nonmotor behavior, and wellbeing. Neurological assessment included general behavior and gait, the latter involving examination of the animal whilst standing and moving, with particular reference to strength and coordination. At the end of the experiment, all animals were killed by sodium pentobarbital overdose (150 mg/kg, i.v.) 1 hr after the last dose of vehicle or ambroxol, and the brains as well as the main organs were removed quickly after death. Each brain was bisected along the midline and the two hemispheres were immediately frozen by immersion in isopentane (−45°C) and then stored at −80°C. Coronal 300 µm‐thick sections were cryostat‐cut and punches of brain and liver tissue were taken for the following regions: midbrain, cortex, striatum, and cerebellum. An average sample size of 6 ± 2 mg was obtained (Bourdenx et al., 2014; Santini et al., 2010).

### 2.3 Enzyme assays

GCase activity and β‐hexosaminidase (HEXB) were measured as described previously (Migdalska‐Richards et al., 2016).

## 3 Results

### 3.1 Ambroxol treatment increased GCase activity in cynomolgus monkeys

**Figure 1:**
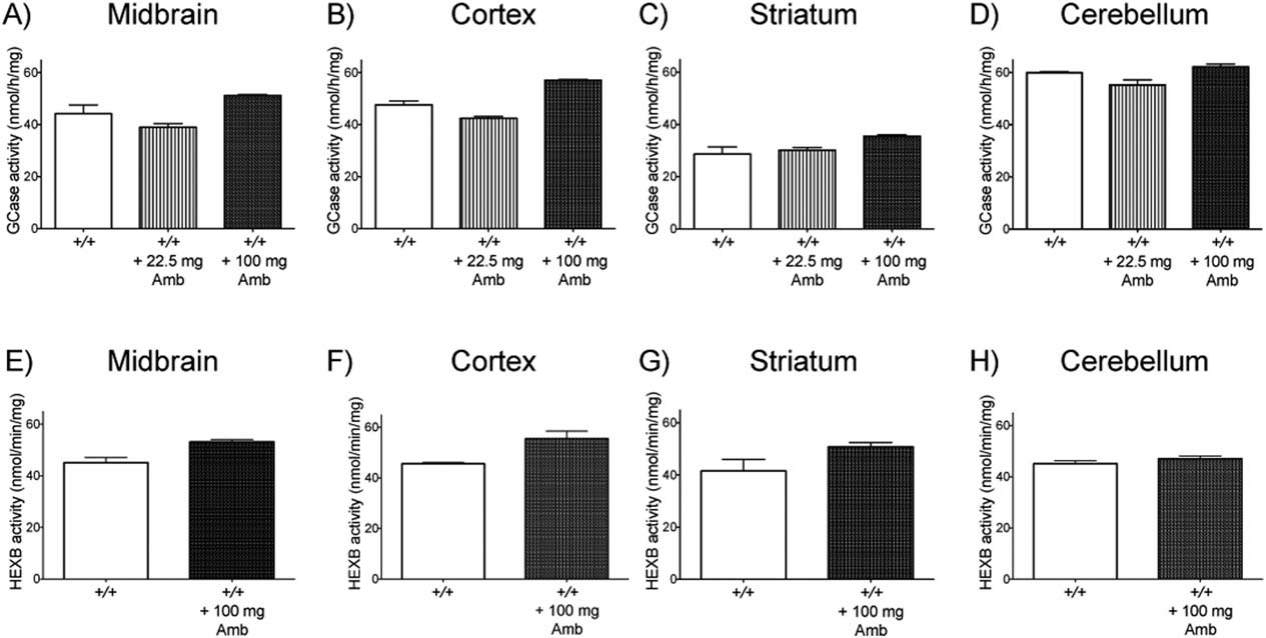
Glucocerebrosidase (GCase) and β‐hexosaminidase (HEXB) activity after daily treatment with different levels of ambroxol. (a–d) GCase activity did not increase with 22.5 mg ambroxol treatment in any region, but increased by about 20% in the midbrain, cortex and striatum and 5% in the cerebellum with 100 mg treatment. (e‐h) HEXB activity was increased by about 20% in the midbrain, cortex, and striatum, but not in the cerebellum, when treated with 100 mg ambroxol

GCase activity was measured in the midbrain, cortex, striatum, and cerebellum of cynomolgus monkeys given 0, 22.5, or 100 mg of ambroxol for 28 consecutive days. Increased activity was found in the midbrain (16%), cortex (20%), and striatum (24%), but not in the cerebellum (only 5%), with 100 mg ambroxol treatment (Figure 1a–d). No increase in GCase activity was observed in any brain region with 22.5 mg treatment (Figure 1a–d).

### 3.2 Ambroxol treatment increased HEXB activity in cynomolgus monkeys

HEXB activity was compared in the midbrain, cortex, striatum, and cerebellum between a cynomolgus monkey given no ambroxol and one given 100 mg of ambroxol for 28 consecutive days. Activity was increased in the midbrain (18%), cortex (22%), and striatum (22%), but not in the cerebellum, of the treated monkey compared with the untreated one (Figure 1e–h).

## 4 Discussion

This study provides the first preliminary data of the effect of ambroxol treatment on GCase activity in different brain regions in wild‐type nonhuman primates.

We observed about a 20% increase in GCase activity in the midbrain, cortex, and striatum of a cynomolgus monkey treated with 100 mg of ambroxol for 28 consecutive days. This finding, although very preliminary and limited to only one animal, suggests that ambroxol might be capable of crossing the primate brain‐blood barrier and increasing wild‐type GCase activity. This observation is in agreement with data obtained from wild‐type mice treated with ambroxol, which also showed about a 20% increase in GCase activity in the same brain regions (Migdalska‐Richards et al., 2016). We also tested the efficacy of 22.5 mg ambroxol at increasing GCase activity in a cynomolgus monkey, but did not observe any changes in the midbrain, cortex, or striatum after 28 consecutive days of treatment. This finding suggests that ambroxol is capable of increasing brain GCase activity only when administered at certain threshold concentrations. This observation is in line with data obtained from wild‐type mice treated with ambroxol, which also demonstrated such pattern of ambroxol efficacy (Migdalska‐Richards et al., 2016).

We also wanted to investigate whether ambroxol has a specific effect on GCase or whether it affects other lysosomal enzymes. We analyzed HEXB enzyme, and observed about a 20% increase in HEXB activity in the midbrain, cortex, and striatum of the cynomolgus monkey treated with 100 mg ambroxol. This observation suggests that ambroxol might also have an effect on lysosomal content. Interestingly, this observation should be compared with data from human PD‐*GBA1* fibroblasts, which showed a decrease in HEXB activity after ambroxol treatment (McNeill et al., 2014), and data from wild‐type and transgenic mice, which showed no changes in HEXB activity following ambroxol treatment (Migdalska‐Richards et al., 2016). This suggests that ambroxol may affect nonhuman primates differently than mice or cell cultures, and indicates that more work is needed to determine the impact of ambroxol on HEXB.

Collectively, our data show that oral ambroxol might be able to increase brain GCase activity in nonhuman primates indicating that ambroxol should be further investigated in the context of clinical trials as a potential treatment for patients with PD and potentially other synucleinopathies (Migdalska‐Richards and Schapira, 2016; Schapira and Gegg, 2013).
